# Aspects and Principles of Material Connections in Restorative Dentistry—A Comprehensive Review

**DOI:** 10.3390/ma15207131

**Published:** 2022-10-13

**Authors:** Zbigniew Raszewski, Dariusz Brząkalski, Łukasz Derpeński, Marek Jałbrzykowski, Robert E. Przekop

**Affiliations:** 1 R&D, SpofaDental, Markova 238, 506-01 Jicin, Czech Republic; 2 Faculty of Chemistry, Adam Mickiewicz University in Poznan, Uniwersytetu Poznańskiego 8, 61-614 Poznan, Poland; 3 Faculty of Mechanical Engineering, Bialystok University of Technology, Wiejska 45 C, 15-351 Bialystok, Poland; 4 Centre for Advanced Technologies, Adam Mickiewicz University in Poznan, Uniwersytetu Poznańskiego 10, 61-614 Poznan, Poland

**Keywords:** adhesion, composite, silane, adhesion, coupling agent, primer, 10-MDP, ceramic, metal, bond

## Abstract

The combination of two dissimilar materials has always been a serious problem in dentistry. In order to meet this challenge, it is necessary to combine both chemical methods (treatment with silanes, (meth)acrylic functional monomers) and the development of the surface of the joined material in a physical way, e.g., by sandblasting with alumina, alumina with silica, acid etching, the use of lasers and other means. The purpose of this literature review is to present all methods of joining dental composites with other materials such as ceramics, metal, another composite material. This review covers articles published within the period 2012–2022 in journals indexed in the PubMed database, written in English and describing joining different dental materials to each other. All the critical steps of new joint preparation have been addressed, including proper cleaning of the joint surface, the application of appropriate primers capable of forming a chemical bond between ceramics, zirconium oxide or metals and alloys, and finally, the application of new composite materials.

## 1. Introduction

In clinical situations, it is a common practice to repair prosthetic restorations. As they are made of a variety of materials very different from each other (metal alloys, ceramics, composite materials), there is no single, universal process for doing so. The first limitation may be a question whether a restoration can be easily removed from the patient’s oral cavity. There are a number of techniques that are a subject of laboratory testing, as they are either very aggressive or technically difficult to execute (acid etch, plasma, etc.), but they cannot be applied to a patient in a dental unit [1]. If it is not possible to debond the prosthetic restoration, it is necessary to perform a repair in the oral cavity. This is often a real challenge for the dentist, especially if the crown or bridge is in the area of the molars: firstly, maintaining adequate isolation from saliva; secondly, appropriate development of the bonded surface so as not to damage the adjacent teeth or soft tissues; lastly, the time of application and polymerization of individual materials [2].

In recent years, a very rapid development and combination of both chemical technology and physical methods in order to obtain an appropriate connection in the oral cavity can be observed [3]. Different dental materials, their biocompatibility and mechanical properties have been a subject of previous review works [4,5,6,7]. The aim of this article is to present the achievements in the field of composite materials with other materials, based on the literature from the last 10 years.

## 2. Methodology

**Figure 1:**
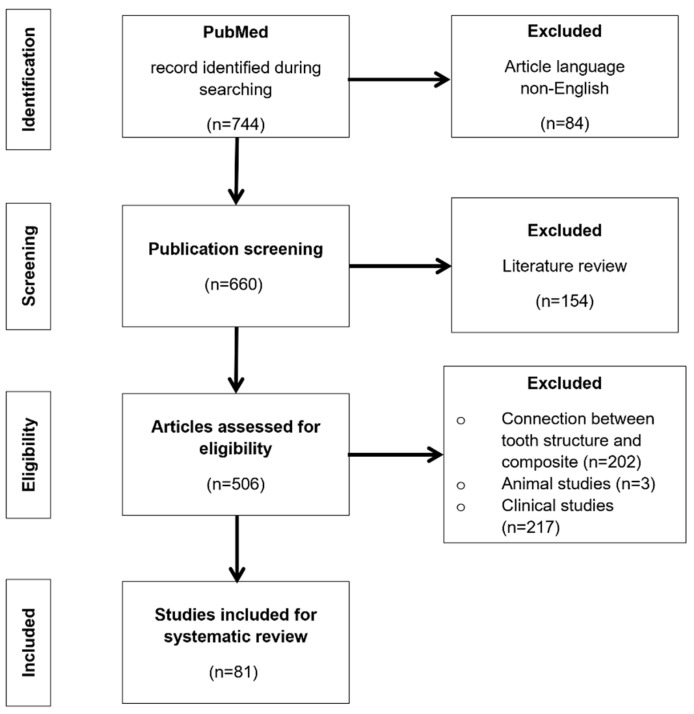
Search and selection methodology for the literature reports covered in this review.

For the review of the literature, PubMed^®^ database was used, with the search words applied that covered adhesion of composite material with metals, zirconium oxide, and composite material. Publications from the period 2012–2022 were reviewed. A total of 744 articles were found. For further evaluation, articles in the English language were qualified, these being 660 works. At a later stage, literature reviews were rejected, giving 506 publications for next evaluation. Of these, 419 were the materials describing the combination of the composite material with enamel and dentin, and three being animal studies. Ultimately, 81 papers published in reputable journals were used to review the literature. The selection criteria are presented in Figure 1.

## 3. Study

### 3.1. Principles of Connections and Connection Strength

Adhesion can be defined as the sum of the chemical and physical forces that represent the molecular attraction between materials in close contact and the mechanical interlocking between the microroughness sites between the joined materials. In dentistry, this can be achieved through the use of appropriate primers, bonds, cements and composite materials, on top of proper physical (grinding, sanding) and chemical (acid etching) preparation of the joined surfaces.

The primer is usually an alcoholic or acetone solution of an acrylic functional monomer having (a) methacrylate group(s) on one reactive site and an acidic or silane moiety on the second one. The bond is a solution of methacrylic resin with large viscosity, dissolved in low viscosity monomers and solvents, also containing a system of catalysts responding to the initiation of polymerization (e.g., camphorquinone for light curing products or amine and peroxide for self-curing). In some commercial products, bond and primer are combined together. There is also a special group of materials known as composite cements, where primer, bond and composite material constitute one product. However, the greatest bonding force is not always required. On one hand, moderate joint strength is sought when fixing orthodontic appliances, which are to be removed after a certain period of time. It is important that the connection strength is not too high, as it could damage the enamel or the entire tooth during appliance removal. Optimum values are therefore around 6–8 MPa. At the other end of the scale, there is the adhesive cementation of bridge crowns or implantological work, where forces of 20–30 MPa are required [8].

### 3.2. Composite Systems for Dental Restorations and Methodology of Establishing a Connection

In clinical use, for fixation and prosthetic restorations, a dentist can choose from three main types of composite systems in terms of curing methodology: light-cured material, self-curing material, and dual cure (light/self-curing). Light polymerized materials can be used in the case of cementation of ceramics, the thickness of which does not exceed 2 mm, so that the light from the polymerization lamp can penetrate through the cured layer and initiate curing reactions in the cement [9].

The first and most important law during all stages of joining two materials is keeping the connection clean. Any loose particles on the surface of the joined material weaken its connection. For any type of repair performed directly in the patient’s mouth, saliva may be the isolating agent. The problem of surface contamination by the saliva or blood is quite widely described in the literature on the subject. Laboratory tests show that if the surface of the tooth becomes contaminated with saliva, the bond strength of metallic brackets bonded to enamel with hydrophilic resin composite can be reduced by 20–30% [10]. In the case of a contaminated surface, it is best to clean it with an ultrasonic cleaner and pure isopropanol and then scrub it again with primer or bonding agent in extraoral conditions. The use of plasma can clean the surface for strong residues, but it is not able to remove silicone-dissolving agents [11]. On the other hand, enzymatic clearing agent was not able to remove contamination form saliva and silicone removing agent effectively and had a negative impact on adhesive properties [12].

For two materials to be permanently joined together, it is important for it to have good wettability, which can be measured by the water contact angle (or with use of other testing liquids, if necessary), the value of which being preferably as close as possible to the value of 0°. Then, the applied substance-bond may spread over the connected surface, filling all the cavities [13]. Another crucial parameter that affects the strength of the connection is the thickness of the bonding layer (primer, bond), the thinner it being, the greater the bonding force [14].

The second very important issue that affects the strength of the bond between the composite material and other surfaces is the time that has elapsed since the material was polymerized. In the case of materials cured with the use of a light or a self-cure, the degree of polymerization of the double carbon–carbon bond in the first few minutes after initiation is within 40–60%. After 24 h, it may be up to 80%. In the case of dual cure cements (light and self-cure), irradiation of such a cement increases the conversion rate of the C=C bond, and thus the strength of the bond with another material [15,16].

Free radicals that are formed under the influence of the light are first formed in the layer closest to the radiation source, and then, by means of propagation reactions, they pass into the deeper layers of the material by the principles of diffusion. Their propagation is strongly influenced by the molecular weight of the reactants and growing polymer network, and reactive and structural characteristics of the material being cured. These can be determined by the shape and volume fraction of the filler, viscosity of the resins, quantity of double bonds and mismatch of refractive index between resin and fillers [8].

The third point that guarantees a proper connection between the two materials is their relative elastic modulus. It is very difficult to obtain a connection between one material which is highly flexible and the other that is very hard (elastic modulus mismatch). In the case of composite materials, it can be a combination at the interface between the dentin, the coronary inlay (made of fiberglass or metal), and cement. This phenomenon was discussed by Ona et al. [17].

When using materials that can be cured with light, the irradiation time plays a very important role, which is properly selected by the material manufacturer. Too short a time, e.g., 8 s compared to the required minimum 20 s, significantly reduced the connection strength. It is known in the dental community that the composite material cannot be ’over- polymerized’, but it cannot be hardened if the light source has insufficient power lower than 350 mW/cm^2^ [18]. The conversion rate of composites can be increased by heating them earlier for 30 min at 40 °C or by using two polymerization methods at the same time. Increasing the temperature and using light curing chamber with inert gas in laboratory conditions (138 °C for 20 min) can increase the bond strength in terms of micro tensile bond strength up to even 70 MP for material Z 100 (3M) [19]. It is necessary to also pay attention that the tip of the light guide of the curing lamp is not contaminated, as it reduces the intensity of the emitted light. The power of the light also decreases with the distance of the light source from the material being cured. Therefore, the tip of the light guide should be kept as close to the surface of the materials being cured as possible.

In the literature reports, the reader can find information stating that in case of the application of incorrect joint establishment methodology or choice of incorrect system components, the joint may become susceptible towards hydrolysis and therefore undergo excessive weakening over time [20].

Commercial bonds contain an alternative substance also a different type of solvent, including acetone, ethanol, and others. After applying such a preparation on the prepared and degreased surface, it is necessary to wait 30–60 s so that the solvent has time to evaporate, and the active monomer has time to form a bond with the surface of the material [21].

During the polymerization of methacrylate resins under atmospheric conditions, an oxygen inhibition layer is formed (because of the greater affinity of the radicals produced with atmospheric oxygen than with each other), which significantly affects the bonding force. To avoid this, a very thin layer of bonding systems or primers should be applied. Secondly, the solvents contained in these materials undergo evaporation, creating a region with a reduced oxygen content directly above the material applied. This problem is not limited to the adhesive film margins exposed to air, but also was evident in adhesive films with entrapped air bubbles inside [22].

### 3.3. Additives for Composite Systems

Producers of dental materials add different kinds of low viscosity monomers to the bonding agents (2-hydroxyethyl methacrylate (HEMA) or triethylene glycol dimethacrylate (TEGDMA)) to reduce the total viscosity of bis-GMA resin (bisphenol A-glycidyl methacrylate). 2-hydroxyethyl methacrylate is a hydrophilic component, while the correct combination of monomers with hydrophilic and hydrophobic properties is crucial, as the evaporation of solvents can lead to the immiscibility between these two types of substances [23]. The non-polymerized layer is not only formed when the composite material comes into contact with atmospheric oxygen. According to various sources, oxygen-free radicals are also formed during the treatment of teeth whitening and may persist in its structure for a period of two weeks. Therefore, it is not recommended to cement new restorations or permanent orthodontic appliances shortly after the whitening treatment [24].

The strength of adhesion may also be influenced by the type of additives, meant to modify composite materials, e.g., in terms of their bactericidal properties, e.g., by means of nanoparticles of silver [25,26], titanium dioxide [27,28], TiF_4_ [29], or zinc oxide [30]. According to K Fatemeh et al., the addition of nano silver will increase the joint strength of the composite material [25,30]. Silver methacrylate and tin di-n-butyldimethacrylateat 1% presented an anti-biofilm effect without altering the mechanical properties [31].

Under laboratory conditions, oral environment is simulated so that its effect on the connection force may be assessed. These tests include, for example, long-term storage in water, artificial saliva, alcohol solutions, etc. This type of testing allows the determination of the resistance of the material to hydrolysis and solubility. The second type of testing is thermal cycling. Testing consists of changing the temperature frequently within the selected limits, e.g., 5–55 °C. The results from this type of test allow researchers to see if the connection may be weakened by expansion and contraction during thermal cycling [32].

## 4. Establishing Connections between Composite Systems and Other Materials

### 4.1. Connection to Zirconium Oxide

In the literature from the last 10 years, over 30% of articles describe the connection between zirconium oxide and various types of cement and composite. This is mainly due to the fact that the all-ceramic restorations made of ZrO_2_ have very good mechanical properties, are translucent and biocompatible. Unfortunately, the main problem arises from the fact that the bonding force between this ceramic and a tooth or other material weakens significantly over time. For this purpose, it is very important to properly prepare the zirconium oxide surface by physically increasing its surface area (develop the surface area for ensuring good mechanical contact), by means of sandblasting with a selected abrasive or grinding with a proper bur. However, not all the procedures tested improved the connection between the composite materials and the zirconium oxide. For example, air blasting with glass beads resulted in a much lower bond strength of the resin cement to all three types of zirconia compared to air blasting with alumina [33]. Tanış et al. obtained the highest bonding strength when using silicon carbide abrasives, cleaned the material in an ultrasonic bath, then applied composite cement to the surface. However, the connecting strength was determined after a relatively short time of 24 h [34]. Al-Akhali et al. observed that the time between sandblasting the zirconium oxide surface and applying the bonding agent should be as short as possible when using 10-MDP (10-Methacryloyloxydecyl dihydrogen phosphate), as only then can the monomers of the primer react properly with zirconium oxide (due to good wettability in meaning of low water contact angle) [35].

Most of the authors agree that increasing the surface area is necessary to establish a proper connection. However, at present, further research is underway on the influence of the size of abrasive particles and the pressure that is used for this procedure. For example, Shimoe et al. used particle sizes (25, 50, 90, 125 µm) and pressures (0.1, 0.2, 0.3, 0.4 MPa) for sandblasting of the two types of zirconia: yttria-stabilized tetragonal zirconia polycrystal (Y-TZP) and ceria-stabilized tetragonal zirconia/alumina nanocomposite (Ce-TZP/A). The obtained results indicate that the particle size does not have a significant influence on the strength of the bond between the composite material and the zirconium oxide, but there was an advantage of using Ce-stabilized ZrO_2_ [36]. The effect of the abrasive action of alumina on the zirconium oxide surfaces and the resulting increase in bonding to the composite material was also investigated by Grasel et al. and Altan et al. [37,38].

Particle size and pressure influence into sandblasting progress of zirconia surface may damage thin layer of crown or bridge; therefore, the use of smaller particles at lower pressure was recommended by Ruales-Carrera et al. (0.15 or 0.25 MPa and an alumina particle size of 50 or 110 µm) [39].

Chen et al. came to the most interesting conclusions, defining the shear bond strength of the connection between composite and zirconium oxide using yttria-stabilized 5Y-TZP tribochemically coated with silica and followed by silanization before and after aging. The obtained results were 13.8 ± 1.4 and 13.2 ± 1.5 MPa, for Lava Plus (3Y-TZP-3% yttrium stabilize) 14.4 ± 1.4 and 13.9 ± 1.6 MPa, for Ceramill Zolid (3Y-TZP) 14.8 ± 1.1 and 13.9 ± 1.5 MPa, respectively. There was no statistical difference between tribochemical silica coating and alumina air abrasion treatments [40,41].

Sandblasting might lead to crystal transition of zirconium dioxide from the tetragonal phase to the monoclinic phase, which reduces the mechanical properties of the material. Therefore, it is necessary to follow the manufacturer’s recommendations regarding pressure and grain size of abrasive materials [32]. Tribochemical silica coating is a more effective method of bonding composite materials with zirconia than the silanization process. This is because the silane does not form a permanent bond with the zirconium oxide surface [42]. The results of research conducted by Cadore-Rodrigues and others, who used alumina particles, as well as alumina with silica coating, for the abrasion of the zirconium oxide surfaces, are promising. When using these materials, the samples stored for 90 days at 37 °C and subjected them to the thermocycling process showed no weakening in the connection at the composite cement–ZrO_2_ interface [43]. Another type of mechanical surface development is the use of silicon carbide bur (SiC grinding bur). In a short period of time, the bond strength is comparable to sandblasting within 32.7 to 41.0 MPa. However, after 150 days, under thermocycling conditions, it was reduced to 21.2 MPa [44].

There is another way to ensure a proper bond between the zirconium oxide and the composite material. It consists of applying silica coating to the connected surface using a radiofrequency magnetron sputtering. According to Uno et al., using gas stream containing 5% oxygen by volume, produced the optimal thickness of silicon oxide layer on the ZrO_2_ surface. Subsequently, a primer containing silane can be applied to the surface prepared in this way, followed by a polymerized composite material. The shear bond strength of the pre-treated zirconia and resin was 35.03 ± 4.97 MPa, which was approximately 3.5 times higher than that of zirconia without any sputtering treatment (9.26 ± 1.21 MPa) [13].

A very important question is how stable over time a connection in discuss will be, because permanent dentures are used in the patient’s oral cavity for an average of 5–15 years. The bond strength of the composite material to zirconium oxide over a period of 5 years has been tested by Aboushelib et al. Specimens whose surfaces were mechanically developed by sand blasting with 50 μm aluminum oxide particles, selective infiltration etching, or fusion sputtering. Then, composite cement was applied to the surface prepared in this way, and the samples were stored in either of five following solutions: artificial saliva, 20% ethanol, 5% NaOH, 4% acetic acid, or 5% phosphoric acid. During storage in these solutions, the weakening of the connection was observed in the order from the greatest to the lowest change: in acid solution, sodium hydroxide solution, and distilled water [45].

Fushiki et al. decided to use the interconnection via an intermediate layer. In this case, feldspar porcelain was fired on the surface of zirconium oxide (Katana), which was then etched with hydrofluoric acid, and a bond and composite material were applied to the surface prepared in this way [46]. A mechanical study confirmed that feldspar porcelain has effectively increased the interconnection strength.

Plasma irradiation, UV treatment, or ceramic primer pre-treatment did not lead to significant increase in bond strength between zirconia and resin composites according to the report of Noro et al. [47]. The approach to using plasma as a tool to increase the adhesion, however, changed over time, because in 2019, several independent reports had already proved that the use of plasma together with self-etching cement has a very large impact on the strength of the bond between the composite material and zirconium oxide [48,49,50].

After the application of argon plasma, the zirconium oxide surface remains at the best activated state for up to about 8 h, during which time it should be cemented from a clinical point of view [51]. Plasma fluorination increases hydroxylation at the surface, making it more reactive, thus allowing for covalent bonding between zirconia surface and resin cement [52].

In the case of zirconium oxide that has not been sintered at high temperature, there is another method for increasing its surface area, presented by Su. It consisted of preparing a suspension containing nano silica and nano zirconium oxide particles in polyethylene glycol. Subsequently, the ZrO_2_ disk is immersed in the suspension for 1 min and dried for 2 h, at room temperature, and then exposed to an oven heating treatment. After the material cooled down, its surface was covered with Monobond N (Ivoclar Vivadent, Lichtenstein). The adhesion force of the composite material to such a surface was 21.28 MPa, and after 6 months of storage in water, it was 16.23 MPa [53].

The dipping method, designed to increase the adhesion, was used by other authors Kim et al. After milling, the zirconium oxide restorations were colored similar to the color of the teeth by applying aqueous salt solutions. It was suggested that these salts can also increase the adhesion between the composite material and the zirconium oxide. The authors concluded that the coloring liquid enhanced the connection between the resin cement and zirconia processed with zirconia primer. In particular, this effect was visible for zirconia colored by immersion in aqueous molybdenum chloride [54].

As mentioned above, a proper connection requires both physical and chemical methods. As an important class of coupling agents, monomers containing methacrylate groups and residues of phosphoric or phosphonium acid should be presented. Chen et al. investigated the effect of 10-methacryloxydecyl dihydrogen phosphate (MDP) concentration in the range of 5–30% to zirconium oxide. The obtained results indicate that 10 wt% MDP appears to be the most optimal concentration of zirconia primers for resin bonding [55]. Functional primers may promote chemical adhesion through forming carboxylate and phosphate salts on Y-TZP. The silane/10 MDP system is very sensitive to pH. According to Ye et al., such mixture can be stored for 7 days, after which there is an internal hydrolysis of the silane, and the bond between the composite and zirconium oxide is reduced with this type of mixture [56].

### 4.2. Connections between Two Composites

There is a consensus among researchers that the adhesion between one composite material that has been in the oral cavity for a long period of time with a second layer of the new material may be a clinical challenge. The new joint created typically has a fracture resistance of approximately 50–60% of the initial value of the composite material. This type of problem is related to the number of non-polymerizable C=C bonds which, under the influence of time elapsed, are reduced significantly, and thus, the new composite material cannot be chemically connected to the older layer. The use of phosphoric acid for this purpose also does not bring the expected results, as it is not able to dissolve the layer of polymerized composite material. According to Najafi et al., the best connection can be created by sandblasting (17.18 ± 1.53 MPa) and there was not a significant difference between the grinding (12.87 ± 3.38 MPa) and laser (11.08 ± 1.37 MPa) treatments for preparing the surface [57].

Espinar-Escalona et al. discussed the usage of different sandblasting particles, alumina and silicon carbide of various mesh sizes, for improving adhesion of brackets to the composite surface [58].

In addition to the traditional methods of preparing the surface of the composite material to be combined with the new layer, in the literature, we can find information about the use of the Er: YAG laser (wavelength of 2940 nanometer, power 2 W). Research carried out by Sobouti F and others indicates that through this method, it is possible to obtain a good connection of 15.38 MPa with a laser power of 2 W and 20.74 with a power increased to 3 W [59].

### 4.3. Connection to Feldspar Ceramics (Silicates)

Feldspar (silicate) ceramics was one of the first materials used in dentistry, due to the good optical properties, but it is not characterized by high fracture resistance (80–120 MPa). It can be used on veneers or single crowns in the area of the anterior teeth. Bridges veneered with feldspar ceramics are used in posterior region and are reinforced with metal substructures which provide adequate resistance to breaking. Since there is a very large difference in the elastic modulus between metal and ceramics, during use, ceramics very often detach from metal. In such a case, it is necessary to perform a repair process. To obtain the correct combination of the composite material with the feldspar ceramics, it is necessary to develop the surface by using hydrofluoric acid. For the proper etching of the ceramic surface, it is necessary to use an stronger medium than phosphoric acid [60]. Hydrofluoric acid is used for this purpose, this substance having glass-dissolving properties, which is a matrix part in feldspar ceramics. The mechanism of action was described by Park et al. [61]. The following chemical reaction equasion is a great simplification of the process:

SiO_2_ + 6HF → 2H_2_O + H_2_SiF_6_

HF acid easily dissociates into hydrogen ion (H^+^) and fluoride (F^−^), affecting the glass surface in two steps. Firstly, acid corrodes the glass surface and thus allows the fluorine ion to pass through the glass. In the glass, the fluoride ion binds to calcium and silicon, and thus interferes with the action of other chemical components of the glass. Silicon molecules in the glass are in the form of a SiO_4_ network linked together by covalent siloxane bonds. In order for these bonds to be broken, it is necessary to break the Si-O-Si connection in the lattice and release the water-soluble silicon salts from the glass, which is performed by HF in the second step of the dissolving process.

Pompeo et al. investigated the use of 10% HF to increase the connection of the composite material with the surface of the steel orthodontic wire. However, according to the authors, this treatment did not improve the connection between these two materials, as this acid does not have the ability to dissolve metal surfaces [62].

According to Komine et al., the correct adhesion protocol of the feldspar ceramics to the composite should include three steps. First, it is necessary to perform sandblasting, then hydrofluoric acid etching and washing with water, and at the end, silane should be applied on the etched surface for creating proper chemical bonding [63]. Some methods of increasing the surface roughness remain controversial at least from the point of view of work safety, e.g., heating the HF acid to 70 °C, which, according to Del Rio et al., increases the bond strength of the composite material to ceramics [64].

In the case of feldspar porcelain, it may be advisable to use 5% additive 3-methacryloxypropyltrimethoxysilane (MPTS or MATMOS) to 1 wt% MDP containing primer. Such treatment allows us to increase the adhesion to 30 MPa [65].

There are reports available that MDP alone does not effectively bond to silica-based ceramics as well as organic silane coupling agents [56].

### 4.4. Adhesion to Non-Precious Metals

Similarly, as in the case of joining one layer of composite material with the other, the factor ensuring proper bonding is the increase in the metal surface area, e.g., by sandblasting or abrasion with a drill [66,67].

The mechanical development of the contact surface area is possible not only by sandblasting but also by the use of retention elements (retention balls, hooks, etc.) pointed into the side of the composite material. This is performed at the stage of the preparation of the wax element, the surface of which is sprinkled with retentive wax beads. After replacing the wax with metal, the obtained retention elements increase the bond strength of the composite to the cobalt chrome alloy as much as two-fold [68].

In the case of a proper connection of the composite material to metal, in addition to the physical development of the surface area, it is necessary to use acrylic functional monomers, which are the active part of various types of self-adhesive cements (SACem). The bonding ability of SACem is basically dependent on the kind of functional monomers that are either phosphoric or carboxylic acid groups. These monomers possess the capability of binding to metal ions through an acid–base reaction [69,70].

Nowadays, titanium is already more often used to replace non-precious alloys in prosthetic applications to avoid allergy, due to its high biocompatibility. The problem is the correct bonding of the composite material to the surface of this metal. Titanium, as a reactive metal, is covered with a thin layer of its oxide upon contact with air. This feature enables the formation of a strong chemical bond between surface Ti and acid acrylic monomers. However, the micromechanical retention by sandblasting plays a bigger role in this connection. According to the thesis put forward by Almilhatti et al., the remnants of the sharp-contoured aluminum particles used in the sandblasting process, remaining in the metal structure, play a very important role as reactive centers for acid acrylic monomers [71].

Another way to increase retention between titanium and composite, has been proposed by Taira et al. A titanium surface was sandblasted with alumina and then etched with 45 wt% H_2_SO_4_ and 15 wt% HCl (H_2_SO_4_-HCl), and the connection strength was over 30 MPa. The authors explain this phenomenon through the use of scanning electron microscopy (SEM) observations revealed that alumina blasting and H_2_SO_4_-HCl etching creates a number of micro- and nanoscale cavities on the titanium surface, which contribute to adhesive bonding [72].

Another connection enhancement mechanism was reported where the Ti surface was treated with polydimethylsiloxane coating and thermal treatment at 800–1100 °C, which provided sufficient resin bonding for clinical services [73].

It was reported that in the case of prosthetic implant structures and restorations made of titanium and cemented to zirconium oxide crowns or bridges, it is necessary to use the sandblasting technique on the interior of the crown made of ZrO_2_ firstly. During the second step, it is necessary to use 10-MDP primer [74,75].

**Figure 2:**
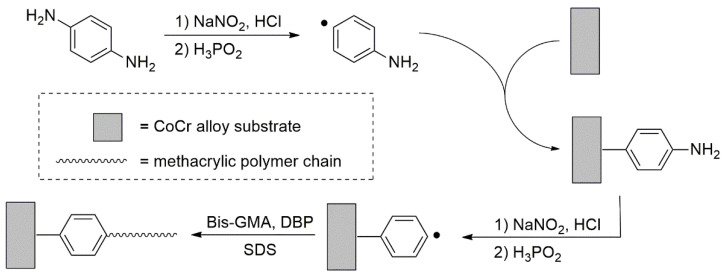
A route for surface treatment of metal with aryldiazonium salts.

In the last 2 years, aryldiazonium salts were used as a material which can create a good connection on the metal surface. They can be easy to prepare and are rapidly reduced to stable radicals that allow strong covalent bonding. The reducing agents activate the aryldiazonium salts and form aryl radicals that covalently bind to the surface (Figure 2). Such bonding is stable up to 200 °C, and resistant to solvents.

In accordance to Oweis et al. p-aminoaniline formed diazonium salt, which upon reduction with H_3_PO_2_ produced a p-aminophenyl radical capable of grafting onto the metal surface of cobalt chromium alloy (CoCr super alloy 6, containing 66% Co, 28% Cr, 6% Mo, and 0.5% others). In the second stage, another free radical is produced in a similar manner on the surface-grafted aromatic group, and finally, with the aid of sodium lauryl sulphate as the surfactant, grafting of bis-GMA onto the modified surface was successfully performed via the aromatic linker introduced in the first step. This reaction is enhanced by the presence of dibenzoyl peroxide (DBP) in the reaction medium (Figure 2). The obtained results of the connection between the surface of the orthodontic bracket and the composite material using this technology were 14 MPa [76].

In the case of metal crowns made of acid-resistant steel, the adhesion of the composite material to their surface can be increased by irradiating the metal surface for 80 min with ultraviolet light and using a 10 MDP primer. The strength of this connection was reported to be of 16.3 MPa. The formation of Cr_2_O_3_ capable of reacting with active monomers was the basis of the explanation for this phenomenon [77].

### 4.5. Adhesion to Precious Metal Alloys

The biggest issues of establishing a proper bond between a composite material and other material are encountered in a scenario when the other material to form a joint with comes from a noble metal alloys class. This is mainly due to the low reactivity of metals such as gold, platinum, or palladium, which do not form an oxide layer on their surface, to which reactive acrylic monomers could bind to. In the first decade of the twentieth century, a compound containing thiophene in its structure was used for this purpose. In the literature, there are methods based on tin plating of noble metals which allows an enhanced mechanical fixation and development of an oxide layer for facilitating establishment of chemical bonds [78].

In the study by Lee et al., three dental gold–palladium–platinum (Au-Pd-Pt), gold–palladium–silver (Au-Pd-Ag), and palladium–silver (Pd-Ag) alloys were used as the bonding substrates after air-abrasion (sandblasting). As coupling agent, solutions of 3-mercaptopropyltrimethoxysilane were used. According to the authors, the strength of the connection was higher than that of the commercial primers for connection with noble metal. These mercaptosilane systems are a promising alternative for improving resin bonding to dental noble metal alloys [79].

### 4.6. Silanes as Adhesion Promoters

Silane-based compounds are commonly used for surface-priming in repairing composite joints. A popular silane agent used for this purpose is 3-methacryloxypropyltrimethoxysilane (MATMOS). One part of this molecule has a methacrylic bond that is able to copolymerize with methacrylic monomers of the composite resin during curing. The other part of this molecule under the presence of aqueous alcohol or water undergoes hydrolysis, upon which formation of free silane groups occurs, which then are capable of reacting with hydroxyl groups on the silica-based ceramic surface to generate strong covalent siloxane (Si-O-Si) linkages. However, these kinds of materials are unstable over time because they can go through dehydration self-condensation, silanols may crosslink into insoluble or non-reactive siloxane oligomers of high molecular weights. The factors that affect the functions of silane include the type of silane, the pH value, the solvent content, the temperature, and the application mode [56]. A parameter considered as a very important one when using silane coupling agents is the time of contact with both the ceramic and composite material. This was investigated by Pilo et al. In their study, samples of the composite material were stored in distilled water for 60 days, and after this period of time, their surface was extended by a mechanical method using tribochemical treatment (CoJet-Sand). The bond strength between the Filteck Suprem XT composite material and the Visio-Bond was approximately 53.9 ± 8.6 MPa; however, it was independent of the silane-based primer exposure time interval applied for the study, which was tested in the range of 1–5 min [80].

The silane alone or in combination with the mechanical increasing of the surface cannot ensure an even and proper bond between the composite cement and the silver–palladium–copper–gold (46% Ag, 20% Pd, 20% Cu, 12% Au) alloy. In this case, it is necessary to use primers based on phosphonic acid monomers [69].

The bonds created by the silanes can undergo the hydrolysis process over time, which is widely described in the literature. However, some silanes can be more resistant to hydrolysis. In the last two years, there have been reports provided of the successful use of 1,2-bis(trimethoxysilyl)ethane (BTME) to bond zirconium oxide to a composite material [32]. Combination of functional (3-acryloxypropyl)trimethoxysilane with bis[3-(trimethoxysilyl)propyl]amine showed superior hydrolytic stability compared with bis-1,2-(triethoxysilyl)ethane (BTEE) [81]. Nowadays, silanes are supplied in a pre-hydrolyzed form in ethanol/water solutions to facilitate their clinical use [76].

### 4.7. Functional Methacrylic Monomers

#### 4.7.1. 10-MDP–10-(methacryloyloxy)decyl Dihydrogen Phosphate

**Figure 3:**
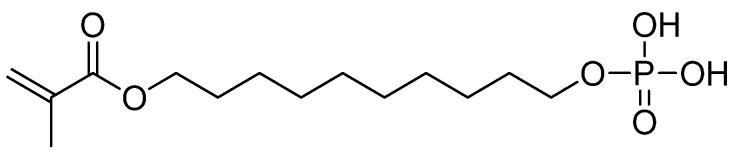
The chemical structure of 10-MDP.

A monomer of this type is capable of establishing a strong chemical interaction with hydroxyapatite, creating a calcium phosphate-related salt. However, impurities and dimers may reduce adhesive forces when using adhesive systems with this functional monomer (Figure 3). The long aliphatic chain between the reactive methacrylate group and the phosphate moiety is responsible for the resistance of this primer to hydrolysis. In the case of non-precious metals, the phosphate functional group may react with surface oxides of titanium, nickel, and chromium to form several species of insoluble and stable salts that form bonds between the composite layer and the metal alloy [20].

The higher viscoelastic properties of the adhesive are suggestive of poor polymerization; therefore, it is used in combination with low viscosity monomers, e.g., HEMA [82].

10-MDP is produced by reacting methacrylic acid with decane-1,10-diol. In the second step, this intermediate is further reacted with POCl_3_. Upon hydrolysis and removal of hydrochloric acid, an ROP(O)(OH)_2_ group is formed at the end of the chain [83,84].

#### 4.7.2. 4-MET–4-(2-Methacryloyloxyethyl) Trimellitic Acid Monoester

**Figure 4:**
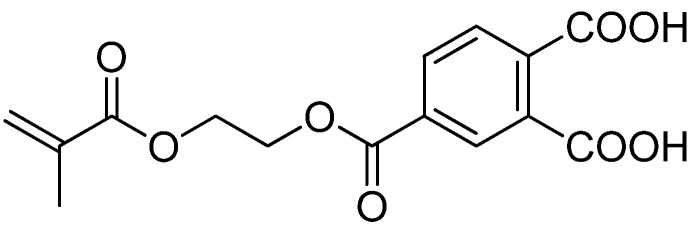
Chemical structure of 4-MET.

An aromatic carboxylic acid monomer may be encountered in some of the bonding systems under the name of 4-MET. Its role is similar to 10-MDP (Figure 4). One of the ends (the carboxylic acid moiety) can be permanently attached to the surface of metals or salts of dentin and the other one (methacrylic group) to composite materials [85].

Other functional monomers used in the production of entanglement between metal and the composite are, e.g., 4-acryloyloxyethyl trimellitate. The two carboxylic groups attached to the aromatic group in it cause improved wettability [71].

## 5. Conclusions

The topic of proper combination of a composite material with other materials used in dentistry remains unresolved to a satisfactory degree and requires further research to give solutions that will provide affordable and approachable methodologies with reproducible and long-lasting effects. In all the articles discussed, the authors note that it is necessary to use various types of methods of increasing the adhesion, both by the development of the surface by sandblasting, acid etching, or the use of laser or plasma treatment. Only on the surface prepared in such a way should the priming agents (functional acrylic monomers, silanes) be applied, increasing the adhesion strength between the two connected materials. Deep understanding of physicochemistry of the used base materials, abrasives promoters, and other activating media is crucial for establishing a strong and lasting joint. Additionally, proper choice of materials in terms of their relative stiffness cannot be neglected in order to prevent joint failure due to fatigue stress. At this point, it is appropriate to mention that a clinically practicing technician shall feel responsible for following the proper steps of the procedure in order to provide long-lasting effects for the sake of the patient’s comfort and life quality Additionally, it should be kept in mind that the scientific and technological progress in the discussed issue is limited by increasing requirements regarding the potential or confirmed toxicity or harmfulness of the substances used. Moreover, the variety of connection variations between different market-available materials present causes additional distraction of efforts in the community to improve the quality of these inter-system connections.

## Data Availability

Not applicable.
